# The Oral Delivery of Water-Soluble Phenol TS-13 Ameliorates Granuloma Formation in an In Vivo Model of Tuberculous Granulomatous Inflammation

**DOI:** 10.1155/2021/6652775

**Published:** 2021-05-14

**Authors:** Elena B. Menshchikova, Peter M. Kozhin, Anton V. Chechushkov, Marina V. Khrapova, Nikolay K. Zenkov

**Affiliations:** Research Institute for Experimental and Clinical Medicine, Federal Research Center for Fundamental and Translational Medicine, Novosibirsk 630117, Russia

## Abstract

The redox-sensitive signaling system Keap1/Nrf2/ARE is a premier protective mechanism against oxidative stress that plays a key role in the pathogenesis and development of various diseases, including tuberculous granulomatous inflammation. We have previously reported that novel water-soluble phenolic antioxidant TS-13 (sodium 3-(4′-methoxyphenyl)propyl thiosulfonate) induces Keap1/Nrf2/ARE and attenuates inflammation. The aim of this study is the examination of the effect of TS-13 on tuberculous granulomatous inflammation. BALB/c mice were administered TS-13 (100 mg kg^−1^ day^−1^) through their drinking water starting immediately after Bacillus Calmette-Guérin (BCG) intravenous injection. Histological changes, production of reactive oxygen species (ROS) (activity of free-radical oxidation processes), and mRNA expression of Nrf2-driven, NF-*κ*B-, AP-1-, and autophagy-dependent signal pathway genes in the liver and peritoneal exudate were evaluated 30 days later. After the 30th day of infection, the activity of the Keap1/Nrf2/ARE system was decreased and its effector genes entailed increasing ROS production in the liver. Therapeutic intervention with TS-13 is aimed at activating the Keap1/Nrf2/ARE system that leads to an increase in Nrf2 and Nrf2-mediated gene expression and a decrease in NF-*κ*B expression. Changes in these pathways resulted in a decline of ROS production and a decrease in the number and the size of granulomas. In total, the results indicate that the Keap1/Nrf2/ARE system can be an effective pharmacological target in host-adjunctive treatment of tuberculosis.

## 1. Introduction

Before the advent of specific antituberculosis drugs (streptomycin, isoniazid, ethambutol, rifampicin, and pyrazinamide) in the middle of the last century, pathogenetic therapy was aimed at reestablishing an ablated immune system and correcting disturbed vital processes; this was the main treatment for patients with tuberculosis. The antimycobacterial therapy demonstrated undoubted success, and resorting to treatment using a specific diet (fish oil or cod liver oil rich in vitamin D) and other methods of pathogenetic therapy have fallen into oblivion or have been put aside. The development of combinatorial therapeutic modalities with the involvement of additional antimycobacterial agents and conventional chemotherapy indicated a breakthrough in therapeutic interventions for tuberculosis, especially in cases of multidrug-resistant tuberculosis. Among the recent drug discoveries, new “host-targeted” adjunct therapeutic strategies have emerged [1–5].

Without a doubt, the additional use of pathogenetic therapy significantly influences tuberculosis treatment and results in better recovery of the functional state in vital systems of the body. In particular, it is the strategy affecting specific signaling systems and key transcription factors, especially redox-dependent (nuclear factor *κ*B (NF-*κ*B) [6–8], activating protein-1 (AP-1) [7], NFE2-related factor 2 (Nrf2)) [8–10] and other key cell processes, including autophagy [4, 6, 11] and lysosomal biogenesis [11].

The key point in *Mycobacterium tuberculosis* survival and persistence in the host is its uptake by macrophages and the subsequent formation of granulomas. The factors involved in the formation and sustainment of tuberculous granuloma are not well understood, but they involve adaptations of both the host and pathogen to a changing microenvironment and redox state. Whether granuloma plays a protective or destructive role in tuberculosis pathogenesis and treatment is the subject of great debates [1, 4, 12]. Besides, the precise mechanisms underlying the redox biology of granulomatous tuberculous inflammation are still obscure. Therefore, the master regulator signaling system Keap1/Nrf2/ARE (Kelch-like ECH-associated protein 1/NFE2-related factor 2/antioxidant respons(iv)e element) can serve as an important target in the pathogenetic treatment of tuberculosis affecting antioxidant protection, xenobiotic detoxification, and activity of reactive oxygen species- (ROS-) generating enzymes [10, 13–15].

In this connection, we have been investigating the impact of sulfur-containing phenolic antioxidant TS-13 on the activation Nrf2 transcription factor [16], on Bacillus Calmette-Guérin- (BCG-) induced granuloma formation and ROS production in mice.

## 2. Materials and Methods

### 2.1. Reagents

**Figure 1:**
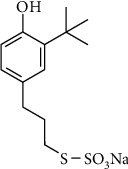
Structure of water-soluble phenolic antioxidant 3-(3′-*tert*-butyl-4′-hydroxyphenyl)propyl thiosulfonate sodium (TS-13).

Luminol was purchased from SERVA Electrophoresis GmbH; 2′,7′-dichlorodihydrofluorescein diacetate and TRIzol Reagent were obtained from Invitrogen; phorbol 12-myristate 13-acetate (PMA) and zymosan were obtained from Sigma-Aldrich; anti-Nrf2 antibodies (ab31163) were purchased from Abcam; RNAlater was purchased from Qiagen; iScript cDNA Synthesis Kit was obtained from Bio-Rad; BioMaster HS-qPCR mix was obtained from Biolabmix; culture media and fetal bovine serum were purchased from Biolot Medical; mounting media were obtained from BioVitrum; BCG vaccine was obtained from Microgen; and sodium 3-(4′-methoxyphenyl)propyl thiosulfonate (TS-13, Figure 1) was synthesized based on 2,6-di-*tert*-butyl-phenоl as described previously [16]. The main substance content in the sample measured by HPLC was 98–99.2%. All other chemicals were analytical grade and were obtained from standard commercial supplies.

### 2.2. Animal Model

The experimental protocol was approved by the Animal Care Committee of the Federal Research Center for Fundamental and Translational Medicine (Novosibirsk, Russia). Male BALB/c mice (weight: 18–22 g; age: 2 months) were purchased from the Federal Research Center Institute of Cytology and Genetics, Siberian Branch of the Russian Academy of Sciences (Novosibirsk, Russia). The animals were housed in an environment where temperature and light were controlled, and food and water were provided *ad libitum*. Mice were injected with 0.5 mg BCG in 0.2 mL saline solution into the tail vein (two groups, 8 animals per group) or 0.2 mL saline solution as a vehicle control (control group, *n* = 6). One group of BCG-infected animals was supplemented with a daily dose of 100 mg kg^−1^ day^−1^ TS-13 dissolved in drinking water for 30 days, while the other groups received water. Mice were sacrificed by cervical dislocation. Peritoneal macrophages were isolated to evaluate the oxidative metabolism of these cells. The livers were quickly removed, weighed, and processed for histological and immunohistochemical examination and preparation of liver homogenates. Samples were collected and stored in RNAlater with subsequent nucleic acid extraction.

### 2.3. Histological Examination

Liver fragments were fixed in 10% neutral formalin, dehydrated in ascending alcohol solutions, and embedded in paraffin. Sections (4-5 *μ*m thick) were stained using the hematoxylin/eosin techniques and studied using light microscopy (AxioImager A1, Carl Zeiss). Specific histochemical staining by the Ziehl-Neelsen stain was used to visualize *Mycobacterium bovis* in the tissues. The numerical density of the granulomas and their diameters were determined by using the morphometry method (AxioVision software, rel. 4.8). These parameters were used as the morphological criteria for tuberculosis activity.

For immunohistochemical study, 3 *μ*m sections were dewaxed and dehydrated, and epitopes were retrieved in citrate buffer solution in a 700 W microwave oven for 20-35 minutes. Endogenous peroxidase was blocked. The samples were incubated with primary anti-Nrf2 antibodies according to instructions. Then, the sections were incubated with horseradish peroxidase (HRP) conjugate (using a Spring Bioscience Corporation detection system) and 3,3′-diaminobenzidine (DAB) substrate and then stained with Mayaer's hematoxylin. Sections were dehydrated using increasing concentrations of ethanol and xylol mounted with synthetic mounting media “Bio Mount,” placed under cover glasses and studied using light microscopy (AxioImager A1, Carl Zeiss).

### 2.4. Activity of Free-Radical Oxidation Processes

#### 2.4.1. Chemiluminescence (CL)

The livers were rinsed with saline, minced with scissors, and homogenized on ice in a Potter-Elvehjem tissue grinder with 5 vol (*w*/*v*) of Hanks' balanced salt solution without phenol red (HBSS) (200 mg/mL). After recording the background CL of the measuring cuvette at 37°C in a chemiluminometer (Photon, Russia) for 2 minutes, 2 mL of liver homogenate was placed in the cuvette and then incubated for 2 minutes; then, the spontaneous CL was measured for 2 minutes. Afterwards, 0.1 mL of 100 nM luminol solution was injected, and the luminol-amplified CL (LACL) was measured for 2 minutes. Then, 0.1 mL of H_2_O_2_ solution was added (final concentration 39.5 mM), and the H_2_O_2_-induced luminol-amplified CL (H_2_O_2_-LACL) was measured. The results were normalized to the CL intensity in control mice.

#### 2.4.2. Flow Cytometry

Peritoneal exudate cells (PECs) were obtained by peritoneal lavage with cold RPMI 1640 medium supplemented with 1% (*v*/*v*) fetal bovine serum and kept on ice until measured. To measure the total ROS production, isolated PECs were incubated for 15 min in 1 mL of HBSS containing 10 *μ*M 2′,7′-dichlorodihydrofluorescein (DCF) diacetate at +37°C. The former is deacylated intracellularly and rapidly oxidized by ROS (predominantly H_2_O_2_) to yield the highly fluorescent product DCF. We measured the intensity of the DCF-dependent fluorescence of PECs stimulated with 100 nM PMA (*λ*_Em_ = 488 nm, *λ*_Ex_ = 520 nm) by using the FACSCalibur (Becton Dickinson, USA) flow cytometer. The gating of the viable macrophages and neutrophils was based on light scattering (forward and side scatter) properties. The results of the cell fluorescence intensity were normalized to the fluorescence in control mice.

### 2.5. Quantitative Real-Time PCR

Total RNA was extracted from tissue using the TRIzol Reagent. Total RNA was converted to cDNA using the iScript cDNA Synthesis Kit. The real-time PCR reactions were carried out using a BioMaster HS-qPCR mix. All reactions were run in triplicate in 96-well plates on a CFX96 system (Bio-Rad), and threshold cycles (Cts) were obtained after 40 cycles. A quantitative assessment was carried out by the delta delta Ct method, and the glyceraldehyde 3-phosphate dehydrogenase (*Gapdh*) gene was used as a reference gene. Primers used in real-time PCR are listed in Table 1.

### 2.6. Statistical Analysis

The Kolmogorov-Smirnov test was used to check whether the variables were normally distributed. For variables with a normal distribution, the one-way ANOVA with post hoc Tukey's test was used for multiple comparisons, and the data are represented as the mean ± SEM. Nonnormally distributed variables were evaluated using the Kruskal-Wallis with post hoc Dunn's test. In case of not normally distributed data the Kruskal-Wallis with post hoc Dunn's test was applied. The data were represented as the median and the lower (Q1) and upper (Q3) quartiles. The relationships between the variables were assessed by Spearman's rank correlation coefficient (*r*_*S*_). Differences between experimental groups and correlations were considered as statistically significant at a *p* value of less than 0.05.

## 3. Results and Discussion

### 3.1. TS-13 Inhibits Granuloma Formation and Enhances Nrf2 Expression (Histological Examinations)

Histological examination showed that disseminated tuberculous inflammation developed 30 days after infection in mice. It was manifested morphologically as BCG granuloma formation in the liver. *M. bovis* bacteria were detected in the foci of the granulomatous inflammation. Granuloma formations around infected macrophages are the hallmarks of chronic mycobacterial infection. The numerical density and diameter of granulomas serve as morphological criteria for the therapeutic efficiency of antituberculosis drugs.

**Figure 2:**
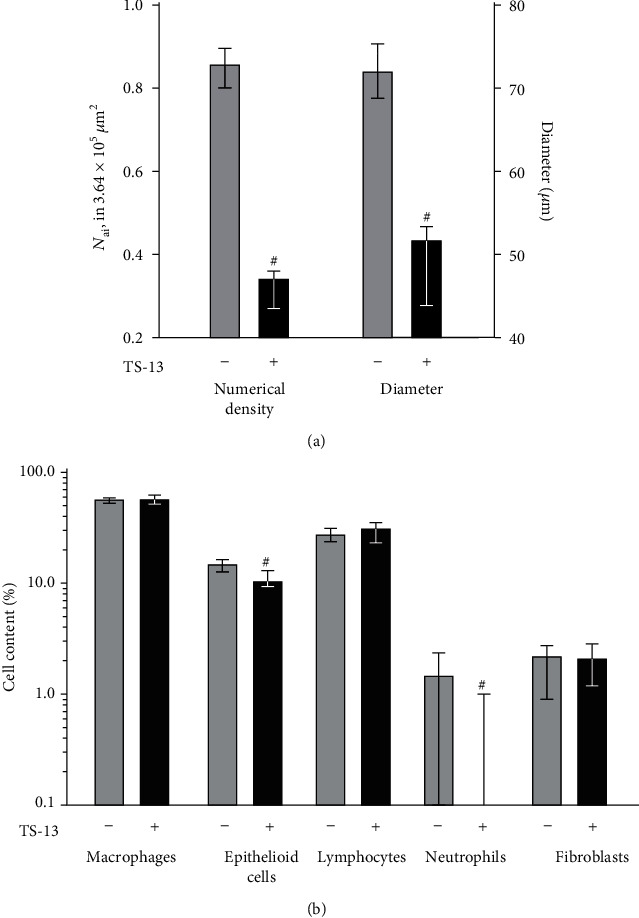
Numerical density, diameter (a), and cellular composition (b) of granulomas in the liver of mice 30 days after BCG infection. Solid bar indicates BCG-infected mice treated with TS-13, and gray bar indicates positive control (BCG-infected mice receiving water). ^#^*p* < 0.05 compared with positive control.

BCG injection induced a marked granulomatous response in the liver of mice with the formation of diffuse, principally perivascular granulomas consisting primarily of macrophages and containing a considerable content of lymphocytes. Morphometric analysis showed that 30 days of TS-13 treatment was followed by a significant 2.5- and 1.4-time decrease in granuloma numerical density and diameter, respectively (Figure 2(a)). Besides, granulomas in the liver of BCG-injected TS-13-treated mice contained significantly less epithelioid cells (by 29.8%) and neutrophils (complete absence) in comparison with BCG-injected TS-13-untreated animals (Figure 2(b)).

**Figure 3:**
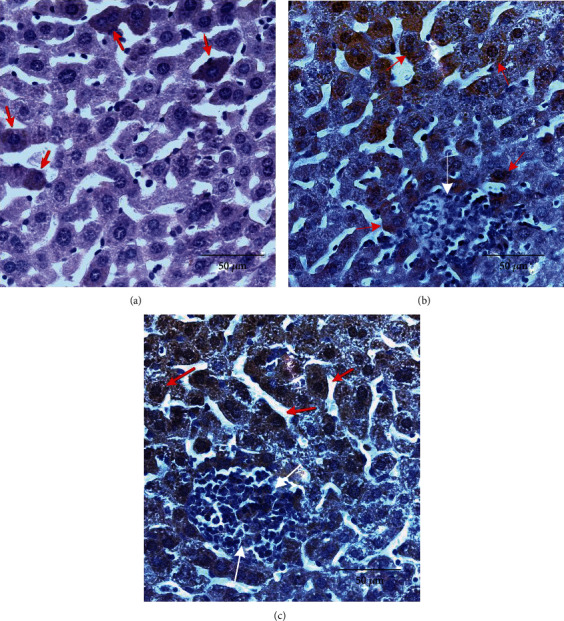
Microphotographs of intracellular Nrf2 expression in mice's liver 30 days after BCG infection (×400). (a) Representative immunohistochemical staining for Nrf2 in livers from control mice. Arrows indicate Nrf2 intracellular localization (cytoplasmic but not nuclear). (b) Representative immunohistochemical staining for Nrf2 in livers from BCG-infected mice. Arrows indicate Nrf2 intracellular localization (cytoplasmic dominant) in hepatocytes (red arrows); granuloma cells are not stained (white arrows). (c) Representative immunohistochemical staining for Nrf2 in livers from BCG-infected mice that received TS-13 in drinking water. Arrows indicate Nrf2 intracellular localization (cytoplasmic and nuclear) in hepatocytes (red) and in granuloma cells (white).

Immunohistochemical analysis of liver samples has demonstrated weak Nrf2 expression with cytoplasmic localization in the control group. In the BCG-infected group, Nrf2 expression was more pronounced in hepatocytes with minimal expression in granulomas. In BCG-infected TS-13-treated mice, Nrf2 was abundant with cytoplasmic and nuclear localization and strong expression in granulomas (Figure 3).

### 3.2. Bidirectional Effect of TS-13 on Free-Radical Oxidation Processes

**Figure 4:**
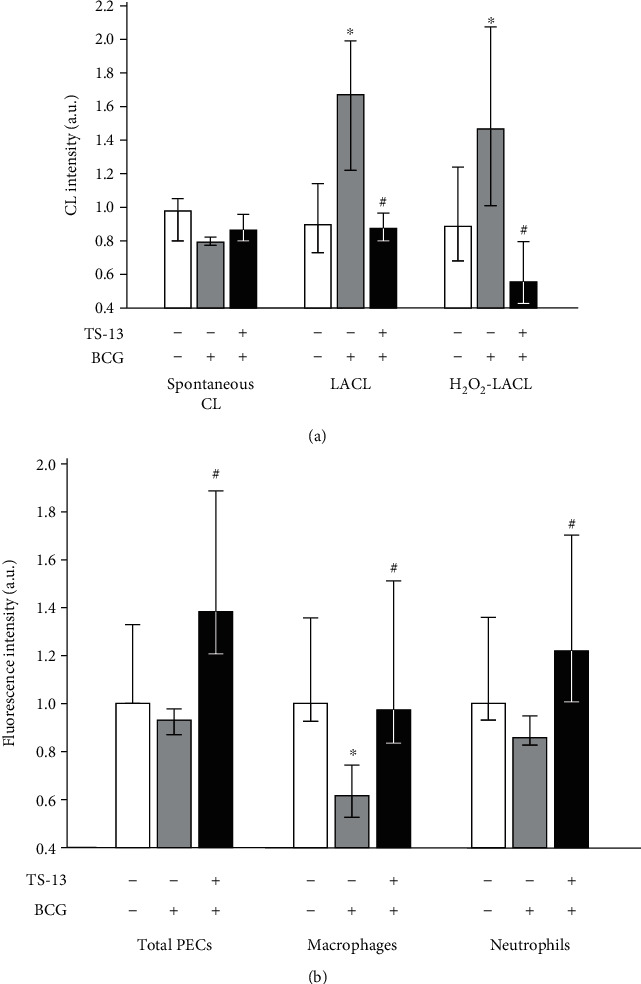
ROS generation in liver ((a) homogenate CL) and by peritoneal exudate cells ((b) DCF-dependent PMA-stimulated fluorescence) 30 days after BCG infection. Open bar indicates negative control (saline-infected mice receiving water), gray bar indicates positive control (BCG-infected mice receiving water), and solid bar indicates BCG-infected mice receiving TS-13 in drinking water. ^∗^*p* < 0.05 compared with negative control; ^#^*p* < 0.05 compared with positive control.

Spontaneous CL is a measure of the generation of lipid alkoxyl and peroxyl radicals, i.e., the intensity of lipid peroxidation [17, 18]. Spontaneous CL levels of liver homogenates did not differ significantly among groups, but when luminol was introduced into a registration system, we observed a significant increase of CL intensity in the BCG-infected group; the same was observed after adding H_2_O_2_ (Figure 4(a)). The levels of liver LACL and H_2_O_2_-LACL intensity of the BCG-infected mice receiving TS-13 were significantly lower in comparison with those of the positive control (BCG-injected mice that received water) (Figure 4(a)).

The PMA-stimulated ROS production by PECs in total and by peritoneal neutrophils from BCG-infected mice was similar to the negative control, whereas DCF-dependent fluorescence of peritoneal macrophages was significantly lower (Figure 4(b)). In BCG-infected TS-13-treated mice, PMA-stimulated fluorescence of total PECs, peritoneal macrophages, and peritoneal neutrophils was statistically higher compared to the corresponding positive control group (Figure 4(b)).

### 3.3. Enhancement of ARE-Driven Pathway by TS-13 Treatment and Effect on NF-*κ*B-, AP-1-, and Autophagy-Dependent Signal Pathways (Gene Expression Analysis)

**Figure 5:**
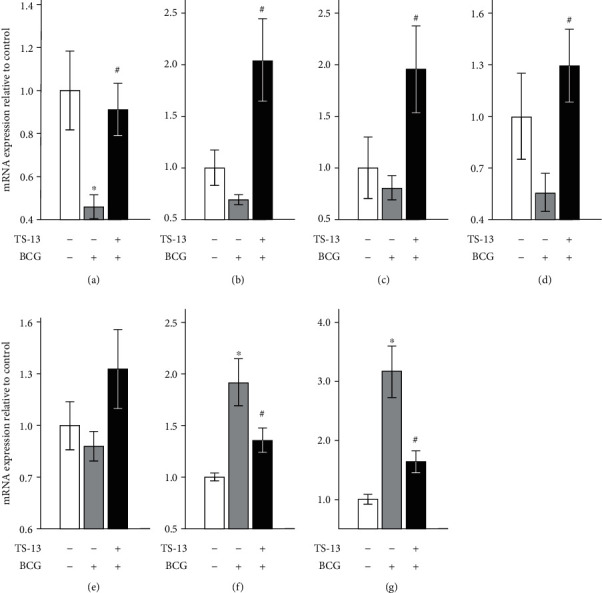
mRNA expression of Nrf2 (а); ARE-driven enzymes NQO1 (b), heme oxygenase 1 (c), and glutathione S-transferase P1 (d); and NF-*κ*B-mediated signal pathway proteins p65 (e), p100 (f), and AP-1 (g) 30 days after BCG infection. Open bar indicates negative control (saline-infected mice receiving water), gray bar indicates positive control (BCG-infected mice receiving water), and solid bar indicates BCG-infected mice receiving TS-13 in drinking water. ^∗^*p* < 0.05 compared with negative control; ^#^*p* < 0.05 compared with positive control.

First, we assessed the effect of TS-13 on the expression of genes involved in redox-dependent signaling pathways, namely, Nrf2-, NF-*κ*B-, and AP-1-mediated (Figure 5). 30 days after BCG infection, mRNA expression of Nrf2 significantly decreased and mRNA content of ARE-driven enzymes (NQO1, glutathione S-transferase P1, and heme oxygenase 1) tended to decrease in the liver of BCG-infected mice in comparison with negative control. An evaluation of mRNA expression of those genes in BCG-infected animals receiving TS-13 with drinking water revealed higher levels of *Nfe2l2*, *Nqo1*, *Gstp1*, and *Hmox1* mRNAs relative to positive control (BCG-infected mice). The expression pattern of p65 (*Rela*), an important subunit of the classical NF-*κ*B pathway, was similar in all 3 groups with a tendency of increasing (*p* = 0.088) in the third group compared to negative control, whereas p100 (*Nfkb2*) mRNA content increased in the BCG-infected group compared to negative control, indicating the alternative activation of NF-*κ*B, which tended to decrease in BCG-infected TS-13-treated mice. Expression of the c-Jun protooncogene (AP-1, *Jun*) was upregulated in the BCG-infected group with a significant decrease in the TS-13-treated group.

**Figure 6:**
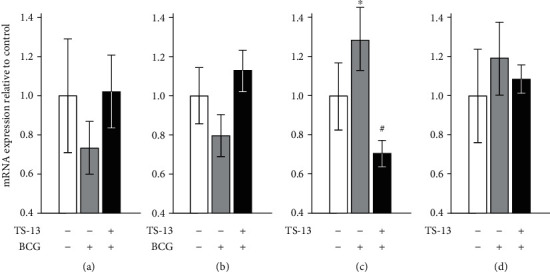
mRNA expression of autophagy effector proteins LC3B (a) and beclin-1 (b) and mRNA expression of lysosomal biogenesis transcription factors TFEB (c) and TFE3 (d) 30 days after BCG infection. Open bar indicates negative control (saline-infected mice receiving water), gray bar indicates positive control (BCG-infected mice receiving water), and solid bar indicates BCG-infected mice receiving TS-13 in drinking water. ∗*p* < 0.05 compared with negative control; ^#^*p* < 0.05 compared with positive control.

Expression of autophagy effector gene *Map1lc3b* (Figure 6(a)) after 30 days of infection did not change in both groups.*Becn1* mRNA expression tended to be more pronounced in the BCG+TS-13 group as compared to positive control (*p* = 0.056) (Figure 6(b)). Expression of lysosomal biogenesis genes *Tfeb* (Figure 6(c)) and *Tfe3* (Figure 6(d)) tended to increase in the BCG group (*Tfeb* mRNA content enhanced significantly). However, *Tfeb* expression decreased to control value in the liver of TS-13-treated mice.

## 4. Discussion

The main purpose of our study is to establish ROS production during experimental tuberculosis in mice and to determine whether induction of the Keap1/Nrf2/ARE signaling pathway could affect granuloma formation.

The lack of Nrf2 nuclear translocation in the BCG-infected group would suggest a defect in Nrf2-mediated antioxidant defense, as previously described [10, 19]. This fact was confirmed by evaluating the expression of the following Nrf2-mediated genes: *Nqo1*, *Gstp1*, and *Hmox1*. Decreased activity of the Keap1/Nrf2/ARE system and its effector genes entails increasing ROS production in the liver. Therapeutic intervention with TS-13 is aimed at activating the Keap1/Nrf2/ARE system that leads to an increase in Nrf2 and Nrf2-mediated gene expression and a decrease in NF-*κ*B expression. The changes in these pathways resulted in a reduction of ROS generation and a decrease in the number and size of granulomas.

We examined the association between the severity of granuloma formation, the intensity of free-radical oxidation processes, and the expression of the Keap1/Nrf2/ARE signaling pathway genes (Table 2). A multivariate regression analysis showed that the free-radical processes involving ROS were important for BCG-induced formation of granulomas in the liver. This is confirmed by our previous assumption of the steady-state hydrogen peroxide concentration increasing in granulomas [20].

Contribution of classical pathway activation of the proinflammatory transcription factor NF-*κ*B was limited. Furthermore, there was an inverse relationship between *Rela* expression and ROS generation and a direct dependence between *Rela* and *Nfe2l2* expression. In contrast, the infection process was accompanied by alternative NF-*κ*B pathway activation that correlated with the H_2_O_2_ production and the c-Jun subunit of transcription factor AP-1 expression.

After 30 days of infection, there was an inverse relationship between the severity of granulomas or ROS formation and *Becn1* expression. This phenomenon is consistent with observations that tuberculous granulomatous inflammation is accompanied by autophagy inhibition, including a decline of *Becn1* expression [5, 10, 11, 21]. At the same time, the elevated levels of expression of lysosomal biogenesis genes *Tfe3* and *Tfeb* positively correlated with expression of *Nfkb2* and *Jun* genes, characterizing the alternative macrophage activation status. The TFEB transcription factor ortholog is a key regulator of immune responses in *Candida elegans*, coordinating expression of genes that are controlled by NF-*κ*B (absent in *C. elegans*) in mammals. Probably, TFEB is one of the oldest high-level regulators of nonspecific immune resistance, which lost the leading role in immune reactions in mammals in connection with the appearance of the more universal regulator NF-*κ*B.

Increased PMA-stimulated ROS production by peritoneal macrophages in BCG-infected TS-13-treated animals might be due to the enhancement of *Nox4* expression as a result of Nrf2 activation under the influence of TS-13 [22, 23]. Mice with partial Keap1 knockout (constitutive activation of Nrf2) is known to have an increased expression of NADPH oxidase homolog NOX4 [15]. PMA stimulation leads to the activation of the NOX4 enzyme and ROS production [23, 24]. At the same time, Nrf2 contributed to the regulation of purine biosynthesis by controlling the expression of genes encoding enzymes in the pentose phosphate pathway, and induction of the Keap1/Nrf2/ARE system might be followed by an increase in the generation of NADPH that is essential for NADPH oxidase-dependent ROS production during a phagocyte respiratory burst [25–27].

## 5. Conclusions

It was shown that the induction of the redox-sensitive signaling system Keap1/Nrf2/ARE in a tuberculous inflammation model reduces the number and size of granulomas and changes the cellular composition of granulomas by decreasing the content of neutrophils (the main destructive factor of inflammation) and epithelioid cells (a factor of the maturity of granulomas). Our results suggest that the Keap1/Nrf2/ARE system could be a plausible and promising pharmacological target for tuberculosis treatment.

## Figures and Tables

**Table 1:** List of forward (F) and reverse (R) primer sequences and TaqMan probes (Pr) used for real-time RT-PCR.

Gene (the encoded protein)	GenBank accession number	Primer sequence
*Gapdh* (housekeeping gene)	NM_001289726.1	F: 5′-AACTTTGGCATTGTGGAAGGGCTC-3′ R: 5′-ACCAGTGGATGCAGGGATGATGTT-3′ Pr: R6G-5′-ATGACCACAGTCCATGCCATCACTGCCA-3′-Q
*Nfe2l2* (transcription factor Nrf2)	NM_010902.4	F: 5′-TGATGGACTTGGAGTTGC-3′ R: 5′-TCAAACACTTCTCGACTTACT-3′ Pr: Fam-5′-TACAGTCCCAGCAGGACATGGATT-3′-Q
*Nqo1* (NAD(P)H : quinone oxidoreductase 1)	NM_008706.5	F: 5′-CGAATCTGACCTCTATGCTATG-3′ R: 5′-TAGAGATGACTCGGAAGGATAC-3′ Pr: Fam-5′-TGAGCTGAAGGACTCGAAGAACT-3′-Q
*Hmox1* (heme oxygenase 1)	NM_010442.2	F: 5′-AGACCGCCTTCCTGCTCAACATT-3′ R: 5′−TGACGAAGTGACGCCATCTGTGA-3′ Pr: Fam-5′-TGAGGAGCTGCAGGTGATGCTGACAGAGGA-3′-Q
*Gstp1* (glutathione S-transferase P1)	NM_013541.1	F: 5′-GATGGAGACCTCACCCTTTA-3′ R: 5′-CCCATCATTCACCATATCCATC-3′ Pr: Fam-5′-CTAATGCCATCTTGAGACACCTTGG-3′-Q
*Rela* (nuclear factor *κ*B p65 subunit)	NM_009045	F: 5′-CAGATACCACCAAGACACAC-3′ R: 5′-ACAAGTTCATGTGGATGAGG-3′ Pr: Fam-5′-AATGGCTACACAGGACCAGGAACA-3′-Q
*Nfκb2* (nuclear factor *κ*B p100 subunit)	NM_019408.3	F: 5′-CTTACTCGCCTCCTTCTAAA-3′ R: 5′-TTTCTTTGGGTATCCCTCTC-3′ Pr: Fam-5′-ATCCATGCAGAGAATGAGGAGCCT-3′-Q
*Jun* (jun protooncogene, AP-1)	NM_010591.2	F: 5′-ACGGAGAAGAAGCTCACAA-3′ R: 5′-GTCCTCTGGGTCAGGAAAG-3′ Pr: Fam-5′-AGGAAATAGGCGAGCGGCTACC-3′-Q
*Becn1* (beclin 1, autophagy related)	NM_019584.3	F: 5′-AGAGGCTAACTCAGGAGA-3′ R: 5′-CCGATCAGAGTGAAGCTATTA-3′ Pr: Fam-5′-AAACTCGCCAGGATGGTGTCTCTC-3′-Q
*Map1lc3b* (microtubule-associated protein 1 light chain 3 beta, LC3B)	NM_026160.4	F: 5′-GAAGTGTACGAGAGTGAGAGAGA-3′ R: 5′-CAACCATTGGCTTTGTTGGAG-3′ Pr: Fam-5′-CGCAGGAGACATTCGGGACAGCAA-3′-Q
*Tfeb* (transcription factor EB)	NM_011549.3	F: 5′-CCCTGTCCACTTCCAGTC-3′ R: 5′-GATACTCCCGAACCTTCTGATG-3′ Pr: Fam-5′-TCCTACCACCTGCAACAGTCCCA-3′-Q
*Tfe3* (transcription factor E3)	NM_172472.3	F: 5′-CACACTGAGTCGTCCACCT-3′ R: 5′-GACAAGTACTGTTTGACCTGCTG-3′ Pr: Fam-5′-AACCCTACACGCTACCACCTGCA-3′-Q

**Table 2:** Relationship (*r*_*S*_) between the features of BCG granuloma, the intensity of free-radical oxidation processes, and the expression of the Keap1/Nrf2/ARE signaling pathway genes.

	*D*	Spontaneous CL	LACL	H_2_O_2_-LACL	PECs	*Nfe2l2*	*NQO1*	*Gstp1*	*Hmox1*
*N*_ai_	0.63^∗^	−0.24	0.59^∗^	0.46	−0.70^∗^	−0.43	−0.69^∗^	−0.63^∗^	−0.52^∗^
*D*		−0.66^∗^	0.73^∗^	0.69^∗^	−0.64^∗^	−0.53^∗^	−0.56^∗^	−0.65^∗^	−0.36
Spontaneous CL		—	−0.27	−0.29	0.08	0.51^∗^	0.47^∗^	−0.10	−0.01
LACL				0.66^∗^	−0.59^∗^	−0.42	−0.57^∗^	−0.74^∗^	−0.36
H_2_O_2_-LACL				—	−0.62^∗^	−0.46^∗^	−0.52^∗^	−0.52^∗^	−0.44
PECs						0.40	0.52^∗^	0.67^∗^	0.27
*Nfe2l2*						—	0.69^∗^	0.06	0.29
*NQO1*								0.30	0.61^∗^
*Gstp1*									0.34

*N*_ai_: numerical density of granulomas; *D*: diameter of granulomas; CL: chemiluminescence; LACL: luminol-amplified chemiluminescence; H_2_O_2_-LACL: H_2_O_2_-induced luminol-amplified chemiluminescence; PECS: DCF-dependent fluorescence of PMA-stimulated peritoneal exudate cells; *Nfe2l2*, *NQO1*, *Gstp1*, and *Hmox1*: mRNA expression of appropriate genes. ^∗^Significant *r*_*S*_ values.

## Data Availability

The data obtained in this study are available from the corresponding author upon request.
